# Supplementary material for: Cysteine-Dependent Conformational Heterogeneity of Shigella flexneri Autotransporter IcsA and Implications of Its Function
Source: Microbiol Spectr. 2022 Nov 14;10(6):e03410-22. doi: 10.1128/spectrum.03410-22 (PMC9769942; doi:10.1128/spectrum.03410-22)
Supplement: Supplemental file 1 — Supplemental material. Download spectrum.03410-22-s0001.pdf, PDF file, 0.5 MB [file spectrum.03410-22-s0001.pdf]

Supplementary Information

Cysteine dependent conformation heterogeneity of *Shigella flexneri* autotransporter IcsA and implications in its function

Jilong Qin[1,2*], Yaoqin Hong[1], Renato Morona[2], Makrina Totsika[1*]

[1]Centre for Immunology and Infection Control, School of Biomedical Sciences, Queensland University of Technology, Brisbane, QLD, Australia

[2]School of Biological Sciences, University of Adelaide, Adelaide, Australia

Correspondence: *Jilong.qin@qut.edu.au *Makrina.totsika@qut.edu.au

Table S1. Strains, plasmids, and oligonucleotides

Bacterial strains

Strains	Description	Source
S. flexneri strains		
2457T	Wild-type *Shigella flexneri* 2a	[1]
Δ*icsA*	*Shigella flexneri* 2a *icsA::tet*	[2]
Δ*ipaD*	*Shigella flexneri* 2a *ipaD::frt*	[3]
Δ*icsA* Δ*ipaD*	*Shigella flexneri* 2a *icsA::tet ipaD::frt*	[3]
Δ*icsB*	*Shigella flexneri* 2a *icsB::kan*	This work
Δ*icsA* Δ*icsB*	*Shigella flexneri* 2a *icsA::tet icsB::kan*	This work
E. coli strains		
DH5α	F– φ80lacZΔM15 Δ(lacZYA-argF)U169 recA1 endA1 hsdR17(rK–, mK+) phoA supE44 λ–thi-1 gyrA96 relA1	Lab stock
TOP10	F–mcrA Δ(mrr-hsdRMS-mcrBC) φ80lacZΔM15 ΔlacX74 recA1 araD139 Δ(ara-leu)7697 galU galK λ– rpsL(StrR) endA1 nupG	Invitrogen
BL21C43(DE3)	F− ompT gal dcm lon hsdSB(rB−mB−) λ(DE3 [lacI lacUV5-T7 gene 1 ind1sam7 nin5]) and two uncharacterized mutations	Lucigen

Plasmids

pIcsA	*icsA* with its native promoter cloned into pBR322	[4]
pBR322	Cloning vector, Amp^R, Tc^R	[5]
pKD46	Temperature sensitive plasmid expressing Red proteins, Amp^R	[6]
pKD4	Plasmid carrying FRT flanked kanamycin resistant cassette, Amp^R, Kan^R	[6]
pIcsB-IpgA	*icsB* and *ipgA* with its native promoter cloned into pSU2718	This work
pSU2718	Cloning vector, Chl^R	[7]
$pIcsA^{737::FLAG}$	FLAG×3 affinity tag inserted at i737 in pIcsA	This work
pIcsA-IcsP	*icsA* and *icsP* cloned into pCDFDuet-1 with FLAG×3 affinity tag inserted at i54 of *icsA*	This work
$pIcsA^{C130S}$	Codon substitution of cysteine to serine at 130	This work
$pIcsA^{C375S}$	Codon substitution of cysteine to serine at 375	This work
$pIcsA^{C379S}$	Codon substitution of cysteine to serine at 379	This work
$pIcsA^{C375S/C379S}$	Codon substitution of cysteine to serine at 375 and 379	This work
$pIcsA^{FLAG-C130S}$	FLAG×3 in-frame addition at i737 in $pIcsA^{C130S}$	This work
$pIcsA^{FLAG-C375S}$	FLAG×3 in-frame addition at i737 in $pIcsA^{C375S}$	This work
$pIcsA^{FLAG-C379S}$	FLAG×3 in-frame addition at i737 in $pIcsA^{C379S}$	This work
$pIcsA^{FLAG-C375S/C379S}$	FLAG×3 in-frame addition at i737 in $pIcsA^{C375S/C379S}$	This work

Oligos

Description	Sequence
NcoI-*icsA* Fwd	CTACGACCATGGCTATGAATCAAATTCACAAATTTTTTGTAATATGACCC
icsA-SalI Rev	CTACGAGTCGACTCAGAAGGTATATTTCACACCCAAAATACC

NdeI-*IcsP* Fwd	CTACGACATATGATGAAATTAAAATTCTTTGTACTTGCAC
icsP-KpnI Rev	CTACGAGGTACCTCAAAAAATATACTTTATACCTGCGG
FLAG i54 addition Fwd	CTCGGGGGGCCAATAGCTTTTGCTACTCCTGACTACAAAGACCATGACGGTGATTATAAAGATCATGACATCGATTACAAGGATGACG
FLAG i54 addition Rev	TGAAAAATGAAGTTCTTGAGTACCCGAAAGCTTGTCATCGTCATCCTTGTAATCGATGTCATGATCTTTATAATCACCGTCATGG
FLAG i737 addition Fwd	CATGACATCGATTACAAGGATGACGATGACAAGCAGATGGATAATCAAGAATCAAAACAG
FLAG i737 addition Rev	ATCTTTATAATCACCGTCATGGTCTTTGTAGTCACTAGTTAGATACCACTTATTGGTATTC
icsB KO Fwd	TGGAGAGTTAATAAAGTATGATCCTCAAAATTAGCAATTTCATTGACGCAGTGTAGGCTGGAGCTGCTTC
icsB KO Rev	AAGCTTATTGATCTGTAATAAGTCGTGAATGTAAAGTTTGGTTCCAAGATATGGGAATTAGCCATGGTCC
icsB-*ipgA* Fwd	AATTTCTAGAAAGAAAATTTGATTATGTTAACAGGCTCAAGTTTC
icsB-*ipgA* Rev	AATTGGTACCCGCGAATTCAACCTGTGGAAG
$IcsA^{C130S}$ Fwd	ACTATTACCATCATTATTATCACCAGCACCACC
$IcsA^{C130S}$ Rev	TCCGGCGGTAATGGTGGTGACTC
$IcsA^{C375S}$/ $IcsA^{C375S/C379S}$ Fwd	TAAATCACCTTCAATAGTTGGTGTG
$IcsA^{C375S}$ Rev	TCCGCTGGTGATTGTACAACTG
$IcsA^{C379S}$ Fwd	ATCACCAGCACATAAATCACC
$IcsA^{C379S}$ Rev	TCCACAACTGTTTCACTATCAGGTAAC
$IcsA^{C375S/C379S}$ Rev	TCCGCTGGTGATTCCACAACTGTTTCACTATCAGGTAAC

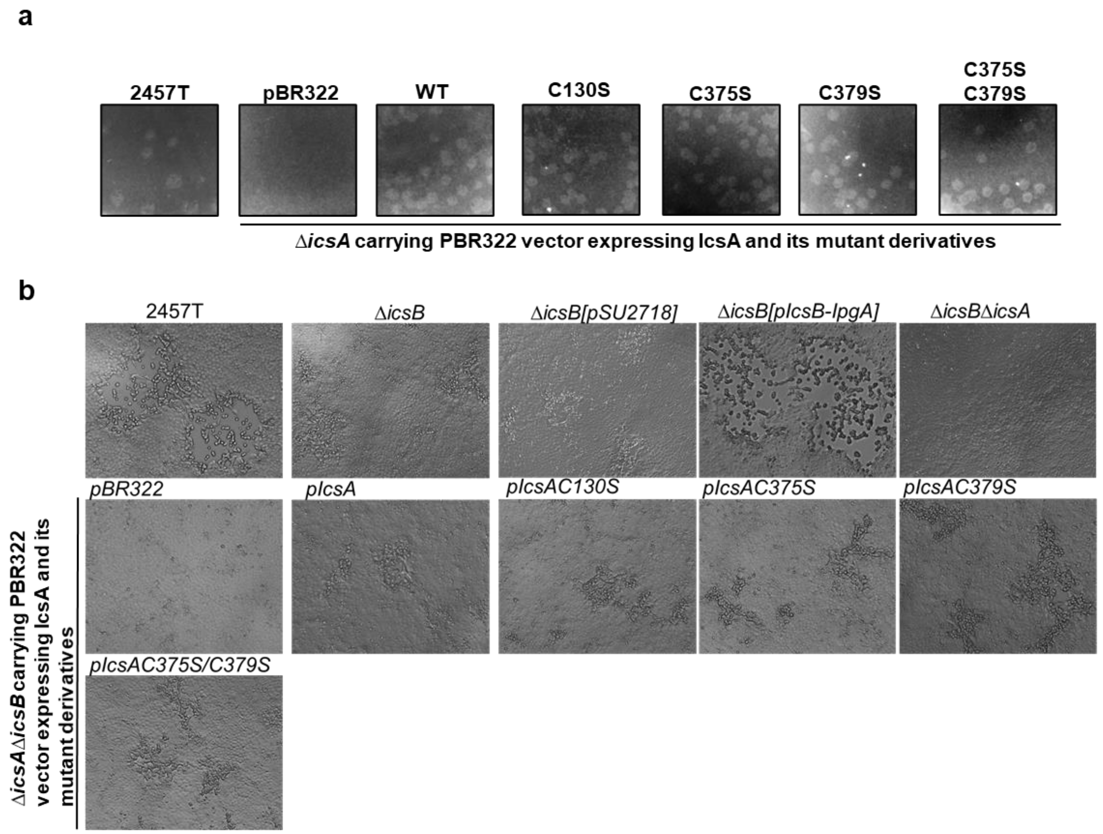

Figure S1. Plaque formation by *S. flexneri* 2457T and its derivatives producing IcsA^{WT} and mutant IcsA proteins with MDCK-2 cells. Representative images were shown, and data was used to generate **Figure 5 d&e** respectively.

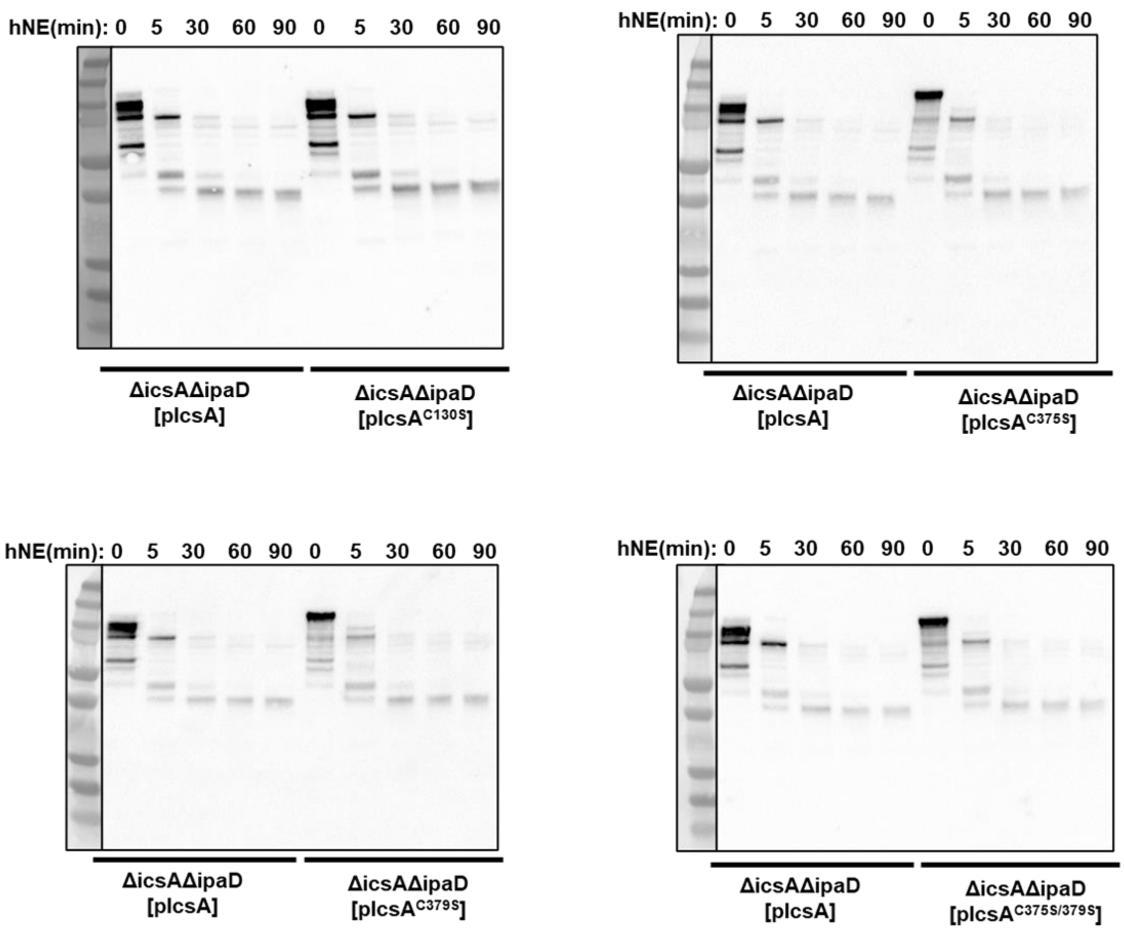

Figure S2. Western immunoblotting with bacterial cells expressing IcsA and its cysteine substitution mutants from 2457TΔipaD treated with hNE. Samples were taken at different time points as indicated above the blot. Samples from bacteria expressing IcsA^{WT} were electrophoresed in parallel (left) with mutants (right) and blotted together to ensure equal exposure for direct comparison. Molecular markers (Precision Plus protein Dual color standards #1610374 BioRad) shown left as 250 kDa, 150 kDa, 100 kDa, 75 kDa, 50 kDa, 37 kDa, 25 kDa, 20 kDa, 15 kDa, and 10 kDa.

References

1 Van den Bosch, L., Manning, P. A. & Morona, R. Regulation of O-antigen chain length is required for *Shigella flexneri* virulence. *Molecular microbiology* **23**, 765-775, doi:10.1046/j.1365-2958.1997.2541625.x (1997).

2 Van den Bosch, L. & Morona, R. The actin-based motility defect of a *Shigella flexneri* rmlD rough LPS mutant is not due to loss of IcsA polarity. *Microb Pathog* **35**, 11-18 (2003).

3 Qin, J., Doyle, M. T., Tran, E. N. H. & Morona, R. The virulence domain of Shigella IcsA contains a subregion with specific host cell adhesion function. *PLoS One* **15**, e0227425, doi:10.1371/journal.pone.0227425 (2020).

4 Morona, R. & Van Den Bosch, L. Multicopy icsA is able to suppress the virulence defect caused by the wzzSF mutation in Shigella flexneri. *FEMS Microbiology Letters* **221**, 213-219, doi:10.1016/s0378-1097(03)00217-9 (2003).

5 Bolivar, F. *et al.* Construction and characterization of new cloning vehicle. II. A multipurpose cloning system. *Gene* **2**, 95-113, doi:https://doi.org/10.1016/0378-1119(77)90000-2 (1977).

6 Datsenko, K. A. & Wanner, B. L. One-step inactivation of chromosomal genes in Escherichia coli K-12 using PCR products. *Proc Natl Acad Sci U S A.* **97**, 6640-6645, doi:D - NLM: PMC18686 EDAT- 2000/06/01 09:00 MHDA- 2000/07/15 11:00 CRDT- 2000/06/01 09:00 AID - 10.1073/pnas.120163297 [doi] AID - 120163297 [pii] PST - ppublish (2000).

7 Martinez, E., Bartolome, B. & de la Cruz, F. pACYC184-derived cloning vectors containing the multiple cloning site and lacZ alpha reporter gene of pUC8/9 and pUC18/19 plasmids. *Gene* **68**, 159-162, doi:10.1016/0378-1119(88)90608-7 (1988).
